# A Complete Hydatidiform Mole Complicated by Theca Lutein Cysts in a Teenager: A Rare Case

**DOI:** 10.7759/cureus.52240

**Published:** 2024-01-14

**Authors:** Saloni, Jyotsna Potdar, Swati M Dahiphale

**Affiliations:** 1 Obstetrics and Gynaecology, Jawaharlal Nehru Medical College, Datta Meghe Institute of Higher Education and Research, Wardha, IND

**Keywords:** theca lutein cysts during pregnancy, gestation, ovarian cyst, pregnancy, molar pregnancy

## Abstract

A hydatidiform mole (HM), often known as molar pregnancy, is a type of prenatal trophoblastic illness that develops in the placenta and has the potential to spread. HMs are caused by genetic issues with either the egg or the sperm. They are typically discovered in the first trimester of pregnancy. Abnormal bleeding is one of the initial symptoms, which can seldom be accompanied by the passage of hydropic villi. Theca lutein cysts, absent fetal heart tones, enlarged uterus more than anticipated for gestational age, pregnancy-induced hypertension in the first trimester, hyperemesis, and increased levels of human chorionic gonadotropin (HCG) for gestational dates are other characteristic symptoms and signs. A rare type of follicular cyst known as a theca lutein cyst is a benign ovarian disease caused by natural overstimulation of follicles, also known as hyperreactio lutealis (HL). This is linked to choriocarcinomas, multiple gestations, and prenatal trophoblastic illness (molar pregnancy). Unless exacerbated by torsion, rupture, or bleeding, the majority of theca lutein cysts are treated conservatively. Theca lutein cysts do not impact the course of pregnancy and spontaneously recede following delivery. However, HL may mistakenly be diagnosed by doctors as a cancer during pregnancy if it has the potential to look like one. Frequently, inappropriate surgical intervention is caused by the fear of failing to diagnose malignancy. These treatments may therefore result in decreased fertility in the future. Here we present a case of a young unmarried female with an HM and cysts.

## Introduction

A proliferative abnormality of placental trophoblastic cells is called a hydatidiform mole (HM). It may result from an abnormal fertilization process and is more prevalent in women under their 20s and beyond the age of 40, or in those who have already given birth to molars. The tumor's origin is unusual because it comes from fetal tissue rather than maternal tissue. Vaginal bleeding, anemia, frequent vomiting, the transvaginal evacuation of grape-like vesicles, an enlarged uterus, hyperthyroidism, and preeclampsia are among the warning signs and symptoms. Based on histopathological and genetic characteristics, HMs are divided into complete and partial forms. Partial moles are often triploid while total moles are typically diploid. An enlarged villous trophoblast with cystic, "swollen," villi is a hallmark of an HM. The development of clusters of vesicles from the metamorphosis of chorionic villi during the second trimester is macroscopically observable. The cytogenetic makeup and microscopic appearance of a whole mole differ from those of a partial mole. In 5-15 percent of cases with entire moles and 1-3 percent of cases with partial moles, progression to gestational trophoblastic neoplasia (GTN) takes place [1]. In 25%-60% of the cases of HMs, ovaries are found to have several theca lutein cysts, a type of bilateral functional ovarian cyst filled with clear, straw-colored fluid. These cysts are a result of either hypersensitivity to human chorionic gonadotropin (beta-hCG) or extreme physiological stimulation known as hyperreactio luteinalis (HL) brought on by high levels of beta-hCG. Multiple theca lutein cysts can be seen on enlarged ovaries on ultrasonography and MRI. Pregnancies complicated by gestational trophoblastic illness have a higher risk of theca lutein cysts, which virtually exclusively arise during pregnancy gestational trophoblastic disease [2,3].

The malignant alteration of trophoblastic cells is known as GTN. The placenta is where all types of GTN develop. Villous trophoblast gives rise to HMs, while interstitial trophoblast gives rise to placental-site trophoblastic cancers. There is a modest probability of resistance in the majority of individuals (approximately 95%) with a molar pregnancy who develop neoplasia. The preferred course of treatment for the majority of these patients is methotrexate or dactinomycin monotherapy. Patients with GTN who are at high risk for developing metastases do so months or years after the causal pregnancy of any kind. Anemia, preeclampsia, hyperthyroidism, respiratory distress, vaginal hemorrhage, and hyperemesis are among the frequently reported late complications of a molar pregnancy, all of which are currently regarded as unusual [4,5]. Most cases of HL occur in the third trimester [6,7]. However, here we report an unusual presentation of a molar pregnancy complicated by HL in the first trimester.

## Case presentation

A 17-year-old unmarried, primigravida girl presented to the Obstetrics and Gynaecology Department with a history of amenorrhea from the last two months, complaining of pain in the abdomen for the last five days. The pain was stretching in character, beginning in the lower abdomen and spreading to the entire abdomen. There were no aggravating or mitigating circumstances, and the patient gave the pain a rating of 7 out of 10. The discomfort was accompanied by nausea and vomiting, for which the patient was started on pantoprazole, ondansetron, and metoclopramide. The patient had never received any sort of hormonal treatment in the past. No noteworthy past medical or surgical history existed.

**Figure 1:**
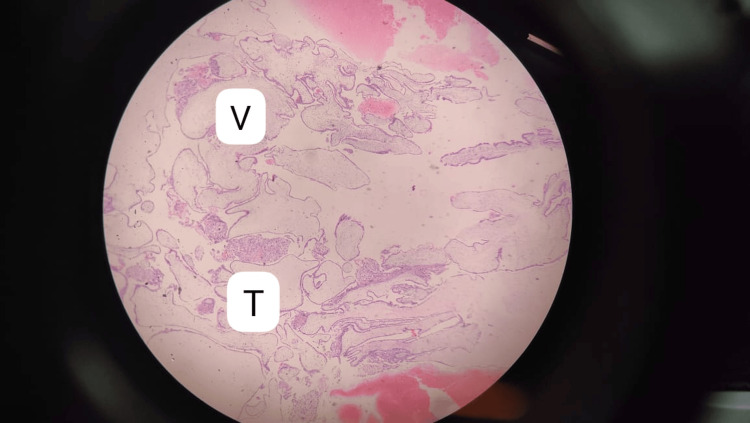
Histology image of the hydatidiform cyst V: Proliferation of villi with edematous stroma; T: Trophoblastic cell proliferation

**Figure 2:**
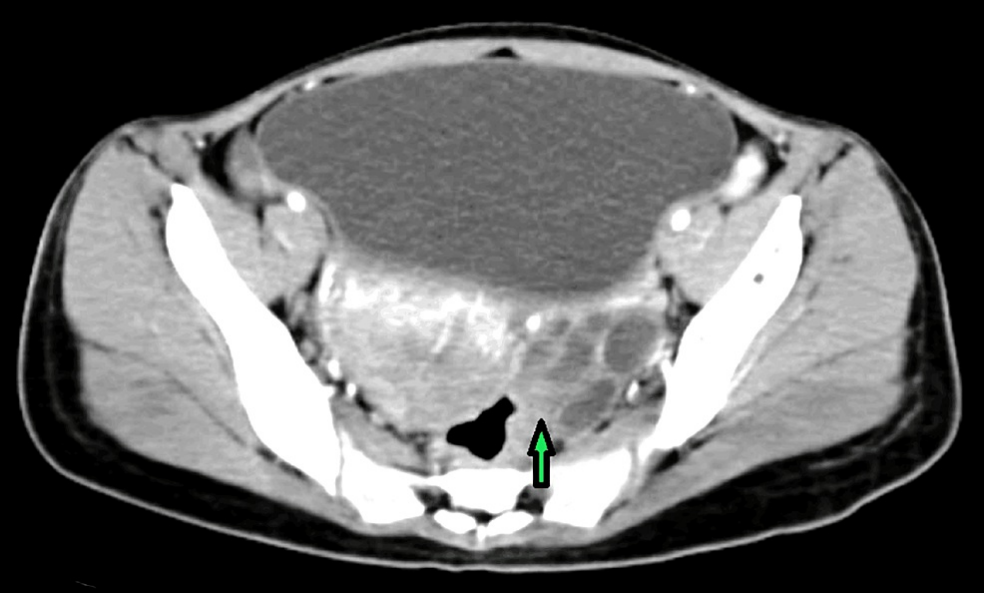
HRCT showing multiple cystic lesions in the ovary (arrow) HRCT: High-resolution computed tomography

The patient's chest X-ray, electrocardiogram, and hemodynamic status (blood pressure 120/80 mmHg, HR 76 bpm) were normal. She was also alert, afebrile, and hemodynamically stable. Her last menstrual cycle was unknown but had occurred two months prior to the positive pregnancy test. She was admitted with a hemoglobin of 8.1 g/dL, mean corpuscular volume of 70 µm, normal platelet counts, urinalysis within normal ranges, random blood sugar of 86 mg/dL, blood group B positive, negative rapid plasma reagin, and seronegative for hepatitis-B surface antigen. The patient was started on injection of methotrexate alternate days, injection of Leucovorin, and mefenamic acid for pain in the abdomen, (the management started because the patient was having symptoms like pain and the value of beta HCG was not returning to normal). The beta human chorionic gonadotropin (bHCG) value was 15000 International Units/liter (IU/L) on examination. A pelvic speculum evaluation revealed no evidence of bleeding in the vagina. However, ultrasonography (USG) revealed a bulky uterus with heterogeneous echogenic endometrial mass with multiple variable-sized cysts and necrotic hemorrhage areas. Contrast-enhanced computed tomography abdomen and pelvis revealed bilateral bulky ovaries showing multiple walled anechoic cysts measuring 12.2×3.5 cm in the right ovary and 8.3×5.6 cm in the left ovary, and uterus 9.2×8.1×5.6 cm, suggestive of a bilateral theca lutein cyst, with minimal endometrial collection (Figure 1 and Figure 2).

**Figure 3:**
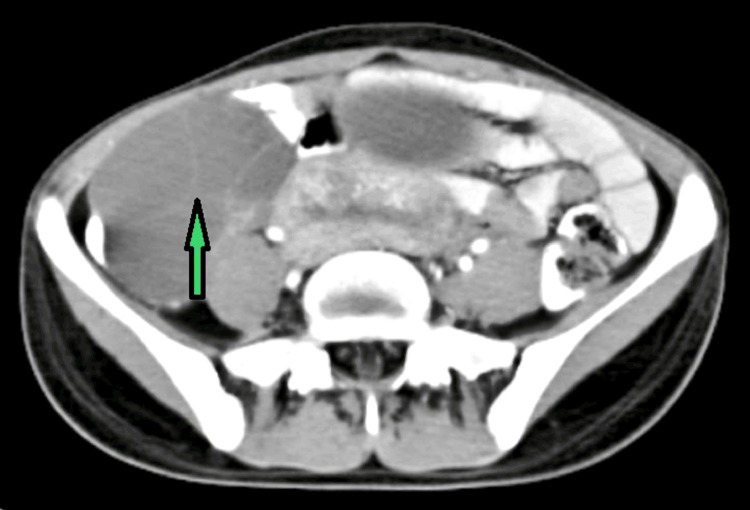
HRCT image showing bulky ovaries (arrow) HRCT: High-resolution computed tomography

Histology revealed the proliferation of villi and trophoblastic cells (Figure 3). From the history and findings, the diagnosis of an HM was clear. Two units of blood were transfused to correct anemia.

Diagnostic and therapeutic suction and evacuation were done under short general anesthesia. The patient was started on methotrexate on alternate days. The values of beta HCG were monitored routinely during follow-up which came down to 8173.3 IU after seven days, 2938.1 IU after 10 days, 485.66 IU on day 14th, and 37 IU after 25 days from the day of evacuation. The bHCG value came to normal and the patient was cured. 

## Discussion

This is a peculiar case of a complete HM with theca lutein cysts, also known as hyperreactio luteinalis. The main cause of HMs is chorion disease. The best classification is that it is a benign neoplasia with malignant potential. The most typical symptoms include bleeding per vagina and pain in the lower abdomen the discharge of grape-shaped vesicles. Complete or partial HMs are the classification for all cases of molar pregnancy. It is typical of complete molar pregnancy to have extensive chorionic villi swelling and diffused trophoblastic hyperplasia without fetus tissues. These patients largely exhibit a diploid karyotype. Prenatal trophoblastic neoplasia, anemia, and structural abnormalities such as syndactyly and cleft lip are also frequently associated with molar pregnancy of partial type [8]. A dense, amorphous mass with many cystic voids that indicate dilated, hydropic villi is typically seen during a molar pregnancy [9].

Past molar pregnancies and the upper and lower limits of maternal age are important risk factors for HMs. Significantly increased HCG levels are frequently linked to preeclampsia, hyperthyroidism, theca lutein ovarian cysts, and hyperemesis gravidarum [10]. In 30-50% of instances, bilateral ovarian theca lutein cysts are observed. After a mole is removed, the American College of Obstetricians and Gynecologists advises that levels of serum-HCG be checked on all patients twice or thrice monthly while the levels are increased and then once every month up to six months until the levels are undetectable [11]. Lutein cysts are a rare variety of follicular cysts that are indicative of an ovarian lesion (benign), which is a physiological overstimulation of follicles [12].

Theca interna layer luteinization and hypertrophy are characteristics of theca lutein cysts. The ensuing bilateral cystic ovaries are varyingly enlarged, with a diameter ranging from 1 to 4 cm and the production of many smooth-walled cysts [13]. The elimination of the choriocarcinoma, the abolition of the molar pregnancy, the cessation of fertility treatment, or delivery is usually sufficient to cause lutein cysts to come to an end on their own naturally. The cysts may take months to go away on their own. Because theca lutein cysts are benign and can disappear after hormonal stimulation is stopped, treatment is conservative [14].

## Conclusions

Theca lutein cyst-complicated molar pregnancy in a 17-year-old girl was properly assessed and treated. This case presents the rare scenario of a girl with an HM during pregnancy which resulted after first intercourse in her teenage years, which is itself a rare condition. A benign disorder called hyperreactio luteinalis results in enlarged ovaries. The importance of recognizing HL is underscored by the fact that incorrect interpretations have led to needless surgery, frequently involving sterilization. Due to HL's self-limiting character, conservative care is recommended, and it is necessary to distinguish HL from other malignant variants. In order to handle these circumstances, serial beta-hCG follow-up is crucial. In situations of molar pregnancy complicated by lutein cysts in young girls, this case emphasizes the value of early identification, appropriate medical therapy, and continuous monitoring.
